# Population structure and spatial distribution of *Mycobacterium tuberculosis* in Ethiopia

**DOI:** 10.1038/s41598-024-59435-3

**Published:** 2024-05-07

**Authors:** Muluwork Getahun, Dereje Beyene, Hilina Mollalign, Getu Diriba, Ephrem Tesfaye, Bazezew Yenew, Mengistu Taddess, Waganeh Sinshaw, Gobena Ameni

**Affiliations:** 1 Ethiopian Public Health Institute, P.O. Box 1242, Addis Ababa, Ethiopia; 2 Department of Microbial, Cellular and Molecular Biology, Addis Ababa University, Addis Ababa, Ethiopia; 3 Aklilu Lemma Institute of Pathobiology, Addis Ababa University, Addis Ababa, Ethiopia; 4 Department of Veterinary Medicine, College of Agriculture and Veterinary Medicine, United Arab Emirates University, Al Ain, United Arab Emirates

**Keywords:** Molecular biology, Infectious diseases

## Abstract

Ethiopia is one of the countries with a high tuberculosis (TB) burden, yet little is known about the spatial distribution of *Mycobacterium tuberculosis* (Mtb) lineages. This study identifies the spoligotyping of 1735 archived Mtb isolates from the National Drug Resistance Survey, collected between November 2011 and June 2013, to investigate Mtb population structure and spatial distribution. Spoligotype International Types (SITs) and lineages were retrieved from online databases. The distribution of lineages was evaluated using Fisher’s exact test and logistic regression models. The Global Moran’s Index and Getis-Ord Gi statistic were utilized to identify hotspot areas. Our results showed that spoligotypes could be interpreted and led to 4 lineages and 283 spoligotype patterns in 91% of the isolates, including 4% of those with multidrug/rifampicin resistance (MDR/RR) TB. The identified Mtb lineages were lineage 1 (1.8%), lineage 3 (25.9%), lineage 4 (70.6%) and lineage 7 (1.6%). The proportion of lineages 3 and 4 varied by regions, with lineage 3 being significantly greater than lineage 4 in reports from Gambella (AOR = 4.37, P < 0.001) and Tigray (AOR = 3.44, P = 0.001) and lineage 4 being significantly higher in Southern Nations Nationalities and Peoples Region (AOR = 1.97, P = 0.026) than lineage 3. Hotspots for lineage 1 were located in eastern Ethiopia, while a lineage 7 hotspot was identified in northern and western Ethiopia. The five prevalent spoligotypes, which were SIT149, SIT53, SIT25, SIT37 and SIT26 account for 42.8% of all isolates under investigation, while SIT149, SIT53 and SIT21 account for 52–57.8% of drug-resistant TB cases. TB and drug resistant TB are mainly caused by lineages 3 and 4, and significant proportions of the prevalent spoligotypes also influence drug-resistant TB and the total TB burden. Regional variations in lineages may result from both local and cross-border spread.

## Introduction

Tuberculosis (TB) is an ancient disease that continues to be a public health challenge^1^. Globally, 30 high TB-burden countries accounted for 86–90% of the estimated global incidence. Ethiopia is listed as one of the 30 countries with the highest prevalence of TB and human immunodeficiency virus (HIV) co-infected TB patients^2^. In 2022, the National TB Program of Ethiopia reported a total of 156,000 TB cases, with an estimated incidence rate of 126 cases per 100,000 population^2^.

TB is caused by nine lineages of *Mycobacterium tuberculosis* (Mtb), of which lineages 1, 2, 3, and 4 contributed significantly to the global TB epidemic^3,4^. Phylogeographic research shows that the geographic distribution of the Mtb lineages varies^4^. Lineages 1 and 3 have been primarily recorded in Asia and East Africa while lineages 2 and 4 occur across the world. The remaining lineages (lineage 5, 6, 7, 8, and 9) are restricted to Africa^4–7^. In addition, the Mtb lineages have variation in transmission success and disease phenotypes^5^. Compared to ancient lineages, "modern" lineages like 2, 3, and 4 are spreading more successfully^8–10^. Additionally, sublineage analysis of lineage 4 identify specific sublineages that have varying geographic distributions^8^. A similar variance has also been seen in lineage 3 clades^6^.

Ethiopia continues to have a high burden of both TB and TB/HIV, even with an annual drop in TB incidence since 1990^11^. Information about the molecular epidemiology of Mtb would support the efforts of the National TB Program. This study utilizes Mtb isolates obtained from the nationwide Drug Resistance Survey (DRS) and evaluate the Mtb spoligotypes and lineages population structure and spatial distribution. We also assess the lineages' hotspot areas.

## Materials and methods

### Study setting and participant enrollment

Ethiopia is a country in East Africa. Six nations border the nation: Sudan, Eritrea, Djibouti, Somalia, Kenya, and South Sudan^12^. Administratively, the country is divided into four levels: regions, zones, woredas (districts) and kebele (wards). The present study utilized Mtb isolates obtained from DRS which were collected from all regions. The DRS was carried out on 32 health facilities, between November 2011 and June 2013. Smear-positive TB patients were the DRS's target populations. A total of 1785 smear-positive TB patients were included. Ninety-seven percent of the isolates (n = 1735) were available for spoligotyping.

### Spoligotyping

All available isolates were undergoing spoligotyping. The spoligotyping was done following the instructions of the manufacturer using a commercially available kit (Qiagen and Sigma)^13,14^. Each run of the spoligotyping has incorporated a positive control (H37Rv and *M. bovis*) and a negative control (water). There was double data entering and cross-checking of any inconsistent readings. An international shared type (SIT) was assigned using the SITVIT2/MIRU-VNTRplus or SpolLineages databases. Lineages were extracted from SpolLineages^15^. Spoligotypes having the same spoligotype pattern were defined as "Cluster" spoligotypes^16^.

### Spatial analysis

The geocode of the health facilities was obtained from the Centers for Disease Control and Prevention of Ethiopia. We utilize ArGIS (version 10.8) for the spatial analysis by taking each health facility as a single unit. The Global Moran’s Index was applied to assess distribution of lineages. Getis-Ord Gi statistic was utilized to identify hotspots. A fixed distance band and a default threshold distance band were used for spatial analysis, which was based on Euclidean distance.

### Statistical analysis

The data was captured and analyzed using SPSS v20 (IBM SPSS Statistics 20). The distribution of lineages and predominate spoligotypes (n > 20)^9^ by drug resistance profiles were evaluated using Fisher’s exact test. Bivariate and multivariate logistic regression models were employed to assess the association of prevalent lineages (lineage 3 and 4, 96.5%) with demographic, clinical, drug resistance and location profiles. The variables with p-value ≤ 0.2 were subjected to multivariable logistic regression models. For the purposes of the logistic regression analysis, the region with the most similar proportion of lineages 3 and 4 to the national average was selected as reference. A p-value of < 0.05 in Fisher’s exact test and multivariate logistic regression was considered statistically significant.

### Ethical approval

This study obtained ethical approval from the Ethiopian Public Health Institute (SERO-59-5-2016) and the Addis Ababa University Ethics Committee Institutional Review Board (SF/MCMB/702/08/2016). We used stored isolates. Personal identifier had not been collected, the DRS enrolled patient using unique survey identification number.

## Results

### Study isolates

A total of 91% (1579/1735) of the isolates, including 68 MDR/RR and 58 INH-resistant Mtb isolates, showed interpretable spoligotype patterns, and each one came from a unique person. Spoligotype and drug susceptibility results were available for 1402 isolates. SITs were retrieved for 88% (1393/1579) of the spoligotypes that were classified into 283 spoligotypes. Ninety percent of the spoligotypes (1423/1579) were grouped into 127 spoligotype patterns with a cluster size of 2–247 (Supplementary Table S1).

### Lineages and spoligotypes identified

Distribution of the lineages and predominate spoligotypes (n > 20) in MDR/RR and INH resistant TB is displayed in Table 1. The five prevalent spoligotypes which were SIT149, SIT53, SIT25, SIT37 and SIT26 accounted for 42.8% of all isolates under investigation. Furthermore, SIT149, SIT53 and SIT21 accounted of 57.3% for MDR/RR and 51.7% of INH resistant TB. The percentage of MDR/RR and INH resistant TB among predominate spoligotypes had significant variation. Lineage information was retrieved for 94.6% (1494/1579) of the spoligotypes (10). *M. bovis* made up 0.13% of the lineages. The Mtb spoligotypes were grouped into four lineages: 1 (1.8%), 3 (25.9%), 4 (70.6%), and 7 (1.6%). The majority of the MDR/RR and INH-resistant TB belonged to lineage 4, which was followed by lineage 3. Within lineages, the percentage of MDR/RR and INH resistant TB had no significant difference (Table 1).

**Table 1:** Distribution of the lineage and predominate spoligotypes in rifampicin and/or isoniazid resistant tuberculosis.

Variables	Spoligotypes/lineage	Number	Percent	MDR/RR	p-value	INH resistant	p-value
Lineages	Total	1494^a^	–	68		58	
	1	27	1.8	3 (4.4%)	0.181	0 (0.0%)	0.703
	3	388	25.9	18 (26.5%)		14 (24.1%)	
	4	1055	70.6	43 (63.2%)		43 (74.1%)	
	7	24	1.6	2 (2.9%)		0 (0.0%)	
	M. bovis	2	0.13	0 (0.0%)		0 (0.0%)	
	Unclassified	83	–^c^	2 (2.9%)		1 (1.7%)	
Predominate spoligotypes	Total	1579^b^		68		58	
	SIT149	247	15.6	23 (33.8%)	< 0.001	18 (31.0%)	0.005
	SIT53	161	10.2	8 (11.8%)		7 (12.1%)	
	SIT25	106	6.7	0 (0.0%)		1 (1.7%)	
	SIT37	93	5.9	0 (0.0%)		0 (0.0%)	
	SIT 26	69	4.4	4 (5.9%)		0 (0.0%)	
	SIT21	49	3.1	8 (11.8%)		5 (8.6%)	
	SIT289	49	3.1	1 (1.5%)		0 (0.0%)	
	SIT52	35	2.2	2 (2.9%)		1 (1.7%)	
	SIT777	27	1.7	0 (0.0%)		1 (1.7%)	
	SIT584	25	1.6	0 (0.0%)		0 (0.0%)	
	SIT3134	23	1.5	0 (0.0%)		1 (1.7%)	
	SIT3137	23	1.5	0 (0.0%)		0 (0.0%)	

^a^The total number represent those isolate with lineage information.

^b^The total number represent those isolate with spoligotype results.

### Spatial distribution of Mtb lineages

Variable proportions of the lineages were reported across regions (Table 2). Dire Dawa was used as a reference since it had the closest proportion of lineage 3 (28.6%) and 4 (71.4%) to the national averages. The bivariate analysis showed that lineage 3 was less likely to be found in Oromia but more likely to be found in places like Gambella, Southern Nations Nationalities and Peoples Region (SNNPR), and Tigray than lineage 4. Only Gambella, SNNPR, and Tigray, however, displayed significant differences on multivariable analysis (Table 3). Accordingly lineage 3 was significantly higher in TB patients from Gambella (AOR = 4.37, P < 0.001) and Tigray (AOR = 3.44, P = 0.001) compared to lineage 4, and lineage 4 was significantly higher in patients from SNNPR (AOR = 1.97, P = 0.026) compared to lineage 3.

**Table 2:** Proportion of the four lineages by regions.

Region	No. of health facilities	Total	L1		L3		L4		L7	
			N	%	N	%	N	%	N	%
Addis Ababa	3	166	0	0.0	51	30.7	108	65.1	4	2.4
Afar	1	62	6	9.7	14	22.6	37	59.7	0	0.0
Amhara	5	124	0	0.0	37	29.8	68	54.8	10	8.1
Benishangul Gumuz	1	19	1	5.3	3	15.8	14	73.7	0	0.0
Dire Dawa	1	90	1	1.1	24	26.7	60	66.7	0	0.0
Gambella	1	106	0	0.0	60	56.6	40	37.7	2	1.9
Harar	1	51	0	0.0	15	29.4	35	68.6	0	0.0
Oromia	11	476	9	1.9	82	17.2	354	74.4	6	1.3
SNNPR	6	318	1	0.3	42	13.2	256	80.5	1	0.3
Somali	1	100	9	9.0	28	28.0	57	57.0	0	0.0
Tigray	1	67	0	0.0	32	47.8	26	38.8	1	1.5

*No.* number, *L* lineage, *SNNPR* Southern Nations Nationalities and Peoples Region.

**Table 3:** Logistic regression analysis of lineage 3 compared with lineage 4 by demographic, clinical variable, drug resistance and regions.

	Variables	L3	L4	COR [95% CI]	p-value	AOR [95% CI]	p-value
Sex	Male	132	427	1.319 [1.034, 1.682]	0.026	1.372 [1.061, 1.774]	0.016
	Female	256	628	Ref		Ref	
Age group	< 15	11	35	0.821[0.344, 1.956]	0.655	NA	
	15–24	132	389	0.886[0.497, 1.579]	0.682	NA	
	25–34	131	335	1.021[0.572, 1.823]	0.944	NA	
	35–44	70	160	1.142[0.620, 2.106]	0.670	NA	
	45–54	24	87	0.720[0.355, 1.460]	0.363	NA	
	55 +	18	47	Ref			
Treatment history	Retreatment	83	157	1.557[1.158, 2.093]	0.003	1.388 [1.000, 1.924]	0.050
	New	305	898	Ref		Ref	
HIV status	Positive	83	144	1.715[1.267, 2.320]	< 0.001	1.313 [0.940, 1.832]	0.110
	Unknown	27	84	0.956 [0.607, 1.506]	0.847	0.940 [0.579, 1.525]	0.802
	Negative	278	827	Ref		Ref	
Regions	Addis Ababa	51	108	1.181 [0.662, 2.106]	0.574	1.437 [0.789, 2.617]	0.236
	Afar	14	37	0.946 [0.435, 2.055]	0.888	1.049 [0.476, 2.314]	0.905
	Amhara	37	68	1.360 [0.732, 2.529]	0.331	1.599 [0.844, 3.029]	0.150
	Benishangul G	3	14	0.536 [0.141, 2.033]	0.359	0.692 [0.180, 2.659]	0.592
	Gambella	60	40	3.750 [2.018, 6.970]	< 0.001	4.374 [2.315, 8.265]	< 0.001
	Harar	15	35	1.071 [0.497, 2.310]	0.860	1.283[0.585, 2.811]	0.534
	Oromia	82	354	0.579 [0.341, 0.985]	0.044	0.689 [0.398, 1.191]	0.182
	SNNPR	42	256	0.410 [0.231, 0.729]	0.002	0.507 [0.279, 0.922]*	0.026
	Somali	28	57	1.228 [0.638, 2.364]	0.539	1.495 [0.762, 2.933]	0.242
	Tigray	32	26	3.077 [1.526, 6.204]	0.002	3.444 [1.685, 7.037]	0.001
	Dire Dawa	24	60	Ref		Ref	
Rifampicin resistance	Yes	18	43	1.151 [0.654, 2.024]	0.626	NA	
	No	326	896	Ref			
Isoniazid resistance	Yes	14	43	0.884 [0.477, 1.637]	0.695	NA	
	No	330	896	Ref			

*NA* not applicable, *G*: Gumuz, *L3* lineage 3, *L4* lineage 4, *HIV* human immunodeficiency virus, *COR* curd odds ratio, *AOR* adjust odds ratio.

*The AOR being in L4 compared to L3 was 1.97.

### Hotspot analysis of Mtb lineages

**Figure 1:**
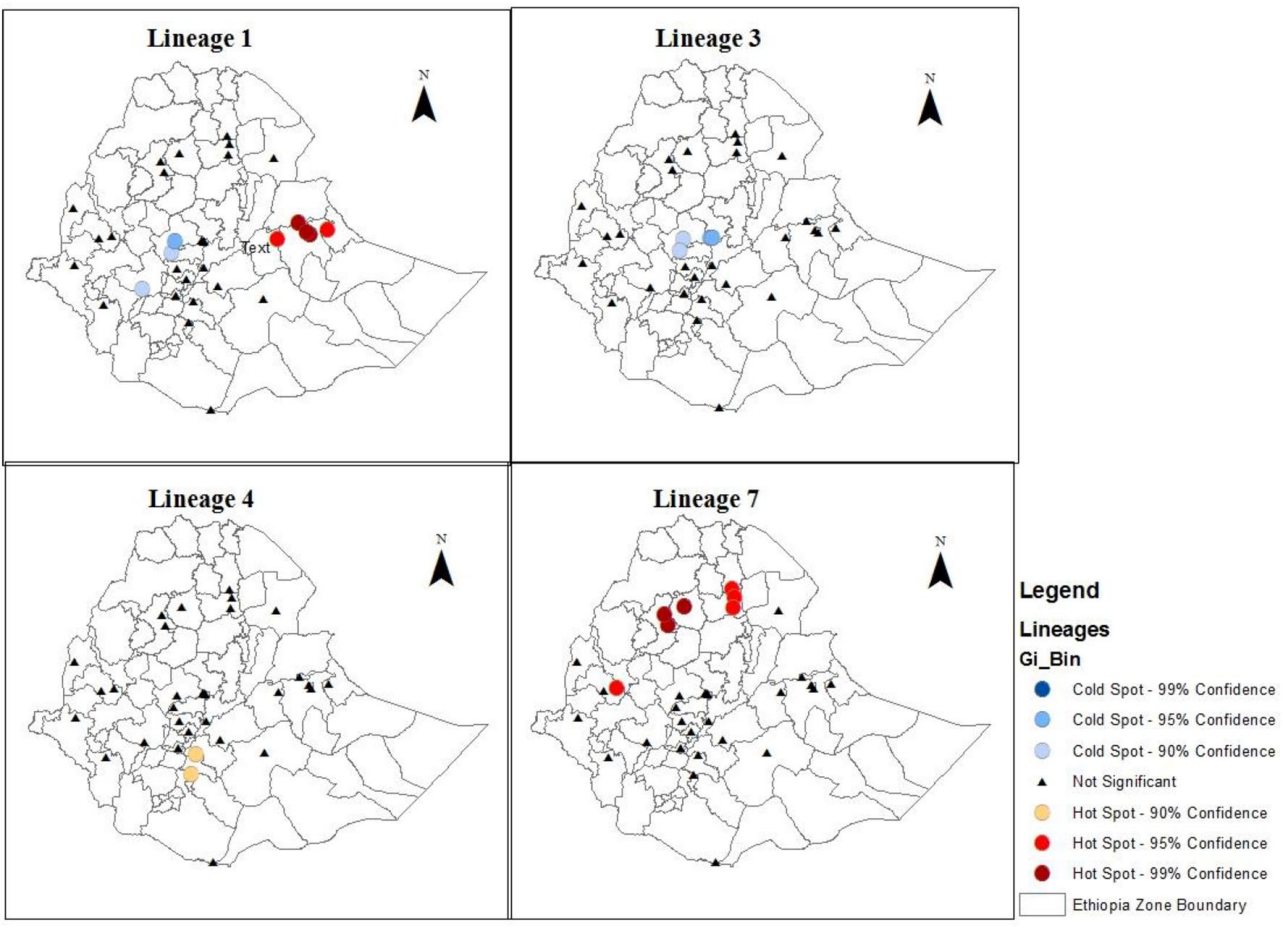
Geographical distribution of hot and cold spot of *Mycobacterium tuberculosis* lineages with confidence interval.

The Global Moran's *I* test revealed that lineage 7 distribution variability was statistically significant (Table 4). Even though, the hot spot was not within a 95% CI, lineage 4 was found to have hotspots in southern Ethiopia. The hotspot analysis is displayed in Fig. 1. Lineage 1 hotspots were identified in eastern Ethiopia while, lineage 7 hotspots were identified in the north and west parts of Ethiopia.

**Table 4:** Global spatial autocorrelation results of Mtb lineages.

Lineages	Moran’s I	z-score	p-value
Lineage 1	0.007158	0.796405	0.425796
Lineage 3	− 0.069844	− 0.739779	0.459434
Lineage 4	− 0.076850	− 0.927205	0.353820
Lineage 7	0.138006	3.516432	0.000437

### Demographic factor associated with dominant lineages

The bivariate analysis of the prominent lineages (lineage 3 and 4) showed that, compared to lineage 4, lineage 3 was more likely to be associated with male, HIV positive, and retreatment TB cases (Table 3). Multivariable analysis, however, revealed that only males exhibited a significant association. In comparison to female patients, male TB patients had increased probabilities of having lineage 3 compared to lineage 4 (AOR = 1.37, P = 0.016).

## Discussion

This study reports on the population structure and spatial distribution of Mtb using isolates collected for the Drug Resistance Survey. Our study showed that 4 out of 9 lineages were circulating in Ethiopia, with lineages 3 and 4 as major lineages that include MDR/RR and INH resistant TB. Furthermore, a noteworthy proportion of MDR/RR (57.8%) and INH resistant (52%) TB is possessed by the three predominant spoligotypes (SIT149, SIT53, SIT21), whilst the five predominant spoligotypes (SIT149, SIT53, SIT25, SIT37, and SIT26) account for a substantial portion of overall TB cases (42.8%). These findings indicate that some spoligotypes have a high percentage which suggests the possible clonal expansion of those spoligotypes in the country. Furthermore, the spatial analysis reveals that the distribution of the lineages vary by region, which is essential knowledge for improving collaborative planning of the TB program activities between the regions of Ethiopia and among neighboring countries.

Examining Mtb strain diversity and distribution allows for a better understanding of TB transmission dynamics and identification of highly transmissible genotypes^6–9^. Certain lineages and spoligotypes have been more prevalent in particular locations; for instance, T2/Uganda II^17^, T3ETH^18^, and EAI2-Manila^19^ have been reported in Uganda, Ethiopia, and the Philippines, respectively. Furthermore, 42–55% of the spoligotypes that have been found can be attributed to 4–5 predominate spoligotypes^18,20–22^. In line with prior studies, our results indicated that 42.8% of the isolates investigated were linked to the five dominate SITs. Additionally, we found that the three predominate spoligotypes have a major share in MDR/RR (57.8%) and INH resistant (52%) TB isolates. Of MDR cases, 60.8% (n = 146/240) in India and 58.2% (n = 100/134) in Zambia were from the three predominate spoligotypes^21,22^. This might be the outcome of the competitive fitness of the strains and the host–pathogen co-evaluation effect, which increase the likelihood that local strains will spread in patient groups within the same nations^5,23^.

The population structures of the Mtb lineages are unique to each country, and the distribution of lineages within a country is also distinct^4,7,17,25^. In the present study, the proportion of lineages 3 and 4 varied among regions, with lineage 3 being significantly greater than lineage 4 in reports from Gambella and Tigray and lineage 4 being significantly higher in SNNPR than lineage 3. Our results are consistent with reports of spatially varied TB lineages within a country. The two prominent lineages in South Africa, lineages 1 and 4, show spatial heterogeneity across province^25^. In comparison to other zones, the Ugandan II family is primarily found in the south-west zone^17^. Furthermore, our data also reflects the dominant lineages in the neighboring countries, such as Sudan (lineage 3), which borders Gambella and Kenya (lineage 4), which borders the SNNPR^26,27^.

Lineage 7 is one of the restricted lineages which is almost exclusively reported in Ethiopia^18,28^. We found that a lineage 7 hot spot has been identified in north and west part of Ethiopia. Prior studies report that lineage 7 is more common in north Ethiopia (13–15.6%) than in other parts of the country (< 0.6%)^19,28–30^, which explains our findings. However, we found 0–1.9% of lineage 7 in the west of Ethiopia, despite the fact that, to the best of our knowledge, lineage 7 has not been documented in this region, which suggests a need of further research. In addition to lineage 7's only reported presence being restricted in Ethiopia, a recent study revealed that lineage 7's reduced protein abundance may contribute to its slower growth and less virulent phenotype^10^. This might contribute to the transmission of lineage 7 in certain locations only, even though the lineage is known for its host–pathogen co-evaluation in Ethiopian TB patients. Our findings indicated that a hotspot for lineage 1 was found in eastern Ethiopia. Previous studies from the eastern part of Ethiopia show that lineage 1 made up 4.7–8.4% of the population, whereas a multicenter analysis found that lineage 1 made up 1.1% of the population^18,29,31^. Furthermore, studies employing sizable data sets revealed that lineage 1/EAI were significantly more common in Somalia (33.63%), which borders the eastern part of Ethiopia^32^. It is possible that local and cross-border transmission in the eastern region of Ethiopia accounts for the increased occurrence of lineage 1.

Gender disparity has been reported in overall TB burden, where the majority (55%) of the global burden, as well as more than 50% of the TB in Ethiopia, occurs in males^2^. The experimental study showed that males acquire Mtb infection far earlier than females due to differences in B cell follicle growth between the sexes^33^. Our results showed that, in contrast to lineage 4, lineage 3 was more common in male patients with an AOR of 1.3 than in female TB patients, which may be partially attributed to male susceptibility. Even though lineage 4 was the predominate lineage in our findings, the gender disparity has not been indicated. There is a possibility that the variation is due to additional underlying patient risk factors, which calls for more carefully monitored research.

This study was subject to limitations. We used spoligotyping to describe the percentage of predominate spoligotypes that may lead to overestimation of the strains in the same categories; hence spoligotyping lacks discriminatory power. Although we include health facilities from every region, the sample we have collected from each region is not representative of the entire region.

## Conclusion

This study reported the population structure of Mtb using samples from all regions of Ethiopia. Our study showed that the Mtb population comprises lineages 1, 3, 4, and 7, with lineages 3 and 4 accounting for the majority of cases of INH-resistant TB, MDR/RR TB, and overall TB. The five predominant spoligotypes account for a significant portion of all TB cases (42.8%), while the three predominant spoligotypes are responsible for a notable percentage of MDR/RR (57.8%) and INH resistant (52%) TB. These might be the result of the possible relative transmissibility advantage of those spoligotypes, which influences drug-resistant TB as well as the total TB burden. The lineage variation by region and the similarity with neighboring countries suggest both local and cross border spread of TB, which is likely to be the result of the bacterial genetic background of the lineages and/or human trafficking.

### Supplementary Information

Supplementary Table 1.

## Data Availability

All the information related to this manuscript is included in this paper and the additional information is provided in Supplementary Table S1.
