# Supplementary material for: Population structure and spatial distribution of Mycobacterium tuberculosis in Ethiopia
Source: Sci Rep. 2024 May 7;14:10455. doi: 10.1038/s41598-024-59435-3 (PMC11076284; doi:10.1038/s41598-024-59435-3)
Supplement: Supplementary file 1 — Supplementary Table 1. [file 41598_2024_59435_MOESM1_ESM.docx]

Supplementary table 1: Clustered stains identified in this study

| SIT | Octal | Lineage by SpolLineages | Total isolates |
| --- | --- | --- | --- |
| 149 | 777000377760771 | L4 | 247 |
| 53 | 777777777760771 | L4 | 161 |
| 25 | 703777740003171 | L3 | 106 |
| 37 | 777737777760771 | L4 | 93 |
| 26 | 703777740003771 | L3 | 69 |
| 289 | 703777740003571 | L3 | 49 |
| 21 | 703377400001771 | L3 | 49 |
| 52 | 777777777760731 | L4 | 35 |
| 777 | 777777777420771 | L4 | 27 |
| 584 | 777775777760731 | L4 | 25 |
| 3137 | 776737777760771 | L4 | 23 |
| 3134 | 777737377720771 | L4 | 23 |
| 142 | 703777700003771 | L3 | 17 |
| 4 | 000000007760771 | L4 | 17 |
| 119 | 777776777760771 | L4 | 16 |
| 50 | 777777777720771 | L4 | 15 |
| 336 | 777776777760731 | L4 | 15 |
| 54 | 777777777763771 | NA | 13 |
| 817 | 777777777420731 | L4 | 13 |
| 302 | 777756777760771 | L4 | 12 |
| 134 | 777777777720631 | L4 | 11 |
| 910 | 700000007177771 | L7 | 11 |
| 1 | 000000000003771 | L3 | 11 |
| 73 | 777737777760731 | L4 | 9 |
| 41 | 777777404760771 | L4 | 9 |
| 47 | 777777774020771 | L4 | 9 |
| 357 | 703777740000771 | L3 | 9 |
| 1729 | 700000004177771 | L7 | 8 |
| Orphan | 006737777760771 | NA | 8 |
| 35 | 777737777420771 | L4 | 8 |
| 699 | 677777777720571 | L4 | 8 |
| 247 | 703777740003471 | L3 | 8 |
| 952 | 603777740003771 | NA | 8 |
| 1134 | 777737777420731 | L4 | 8 |
| 42 | 777777607760771 | L4 | 7 |
| Orphan | 776737677760771 | L4 | 7 |
| 2359 | 703677740003171 | L3 | 7 |
| 345 | 777000377760731 | L4 | 6 |
| 40 | 777777377760771 | L4 | 6 |
| 46 | 777777770000000 | L4 | 6 |
| Orphan | 000000017420771 | NA | 5 |
| Orphan | 773777776000771 | NA | 5 |
| Orphan | 776737737760771 | L4 | 5 |
| 137 | 777776777760601 | L4 | 5 |
| 3325 | 776737407760771 | L4 | 5 |
| 602 | 777777770000771 | NA | 4 |
| 56 | 777737770000000 | NA | 4 |
| 1675 | 703367400001771 | L3 | 4 |
| 121 | 777777775720771 | L4 | 4 |
| 48 | 777777777413731 | L1 | 4 |
| 231 | 777777757760700 | L4 | 4 |
| 764 | 777757777720771 | L4 | 4 |
| 36 | 777737777720771 | L4 | 4 |
| 118 | 777767777760771 | L4 | 4 |
| 20 | 677777607760771 | L4 | 4 |
| Orphan | 777000377160771 | L4 | 3 |
| 1163 | 677737777760771 | L4 | 3 |
| 3 | 000000007720771 | NA | 3 |
| 1547 | 777727777760771 | L4 | 3 |
| 3133 | 677777777420731 | L4 | 3 |
| 2793 | 400000757760771 | L4 | 3 |
| Orphan | 703777700001771 | L3 | 3 |
| 309 | 703767740003171 | L3 | 3 |
| 883 | 777777754020771 | L4 | 3 |
| 462 | 777777777560771 | L4 | 3 |
| Orphan | 737377377760771 | L4 | 3 |
| 343 | 700000007175771 | L7 | 3 |
| 2683 | 703777600003171 | L3 | 3 |
| Orphan | 777777377420771 | L4 | 3 |
| 3315 | 376777737760771 | L4 | 3 |
| Orphan | 707737747413771 | L1 | 3 |
| 798 | 437777777760771 | L4 | 3 |
| 1812 | 777777637720771 | L4 | 3 |
| 591 | 777777757413771 | L1 | 3 |
| Orphan | 340037777760760 | L4 | 3 |
| Orphan | 777777457413371 | L1 | 3 |
| Orphan | 760000777760771 | L4 | 3 |
| 1200 | 703777747777771 | L3 | 3 |
| 2570 | 777777337760771 | L4 | 3 |
| 141 | 703767740003771 | L3 | 3 |
| Orphan | 557767777740771 | L4 | 2 |
| 156 | 776177777760771 | L4 | 2 |
| 217 | 777736777760771 | L4 | 2 |
| Orphan | 777777742020731 | L4 | 2 |
| 281 | 777775777760771 | L4 | 2 |
| Orphan | 777757477760731 | L4 | 2 |
| Orphan | 737776774720171 | L4 | 2 |
| 943 | 777777757760731 | L4 | 2 |
| Orphan | 776737767760771 | L4 | 2 |
| Orphan | 777777747420771 | L4 | 2 |
| Orphan | 777737777420531 | L4 | 2 |
| 100 | 777777777773771 | NA | 2 |
| 1198 | 703737740003171 | L3 | 2 |
| Orphan | 743777777420731 | L4 | 2 |
| 1312 | 703777740003131 | L3 | 2 |
| Orphan | 677777277413771 | L1 | 2 |
| Orphan | 777737737760770 | L4 | 2 |
| 2928 | 503367400001771 | L3 | 2 |
| 1877 | 737377777760771 | L4 | 2 |
| 708 | 737776777760771 | L4 | 2 |
| 1967 | 703777740000031 | L3 | 2 |
| 442 | 777737377760771 | L4 | 2 |
| 504 | 777737737760771 | L4 | 2 |
| 586 | 0000007720631 | NA | 2 |
| 262 | 774777777420771 | L4 | 2 |
| 44 | 777777757760771 | L4 | 2 |
| 17 | 677737607760771 | L4 | 2 |
| Orphan | 777000077760771 | L4 | 2 |
| 356 | 703777600001771 | L3 | 2 |
| 196 | 677777777760771 | L4 | 2 |
| 3450 | 777777405720771 | L4 | 2 |
| 2551 | 777777607760701 | L4 | 2 |
| Orphan | 703777740003431 | L3 | 2 |
| Orphan | 700000007173771 | L7 | 2 |
| 354 | 777777777403731 | NA | 2 |
| Orphan | 777737777740731 | L4 | 2 |
| Orphan | 677777577420731 | L4 | 2 |
| 726 | 777737747413771 | L1 | 2 |
| Orphan | 700000007760771 | L4 | 2 |
| 3142 | 777347777760661 | L4 | 2 |
| Orphan | 777760000060771 | L4 | 2 |
| Orphan | 477777376413771 | L1 | 2 |
| Orphan | 703777340003171 | L3 | 2 |
| Orphan | 703677740003571 | L3 | 2 |
| 1949 | 703777700000771 | L3 | 2 |
| Orphan | 557000377740771 | L4 | 2 |
| 11 | 477777777413071 | L1 | 2 |

NA: not available, L: lineage
